# Development and Evaluation of Statistical Models Based on Machine Learning Techniques for Estimating Particulate Matter (PM_2.5_ and PM_10_) Concentrations

**DOI:** 10.3390/ijerph19137728

**Published:** 2022-06-23

**Authors:** Wan Yun Hong, David Koh, Liya E. Yu

**Affiliations:** 1 Faculty of Integrated Technologies, Universiti Brunei Darussalam, Gadong BE1410, Brunei; 2 PAPRSB Institute of Health Sciences, Universiti Brunei Darussalam, Gadong BE1410, Brunei; ephkohd@nus.edu.sg; 3 SSH School of Public Health, National University of Singapore, Singapore 117549, Singapore; 4 Department of Civil and Environmental Engineering, National University of Singapore, Singapore 117576, Singapore; liya.yu@nus.edu.sg

**Keywords:** PM_2.5_, PM_10_, statistical modelling, machine learning, Brunei Darussalam, Singapore

## Abstract

Despite extensive research on air pollution estimation/prediction, inter-country models for estimating air pollutant concentrations in Southeast Asia have not yet been fully developed and validated owing to the lack of air quality (AQ), emission inventory and meteorological data from different countries in the region. The purpose of this study is to develop and evaluate two machine learning (ML)-based models (i.e., analysis of covariance (ANCOVA) and random forest regression (RFR)) for estimating daily PM_2.5_ and PM_10_ concentrations in Brunei Darussalam. These models were first derived from past AQ and meteorological measurements in Singapore and then tested with AQ and meteorological data from Brunei Darussalam. The results show that the ANCOVA model ($R^{2}$ = 0.94 and RMSE = 0.05 µg/m^3^ for PM_2.5_, and $R^{2}$ = 0.72 and RMSE = 0.09 µg/m^3^ for PM_10_) could describe daily PM concentrations over 18 µg/m^3^ in Brunei Darussalam much better than the RFR model ($R^{2}$ = 0.92 and RMSE = 0.04 µg/m^3^ for PM_2.5_, and $R^{2}$ = 0.86 and RMSE = 0.08 µg/m^3^ for PM_10_). In conclusion, the derived models provide a satisfactory estimation of PM concentrations for both countries despite some limitations. This study shows the potential of the models for inter-country PM estimations in Southeast Asia.

## 1. Introduction

Atmospheric air pollution has been a concern globally for decades [1] because it is a major environmental risk to health. In 2016, ambient air pollution was estimated to cause 4.2 million worldwide deaths annually due to stroke, heart disease, lung cancer as well as acute and chronic respiratory diseases, including asthma [2]. Air pollution exposes people to particulate matter (PM) and other air pollutants such as ground-level ozone (O_3_), nitrogen dioxide (NO_2_) and sulphur dioxide (SO_2_). These air pollutants have strong evidence of health effects [3]. Air pollution can also cause various harmful environmental effects such as global warming, climate change, acid rain, eutrophication, haze, ozone depletion as well as crop and forest damage [4]. According to the World Health Organization (WHO), air pollution in the Southeast Asia region is among the highest in the world [5]. The air quality in Southeast Asian countries including Brunei Darussalam and Singapore have been seasonally affected by transboundary smoke haze due to land and forest fires in the region [6,7,8], normally from August to October during the Southwest monsoon period [9]. Precautionary measures to minimize the exposure of air pollutants on individuals could be taken if the air quality and the air pollutant concentrations in the region were known to the community. However, not all countries have long-term regular air quality monitoring data of desired spatial and temporal density-frequency to sufficiently communicate, alert and protect the public. This shows that there is a need to estimate the air pollutant concentrations in regions or countries that do not have the monitoring data. To address these needs, this study will focus on PM, one of the major air pollutants worldwide and a dominant air pollutant during smoke haze with 94% of airborne particles affected by burning smoke of sizes ≤ 2.5 µm (PM_2.5_) [10].

With complex factors of varied emission strength, composition of sources and changeable meteorological conditions, a promising effective way to estimate air pollutant concentrations is by using statistical models based on machine learning (ML) techniques. This approach could overcome the limitation of traditional deterministic models by accounting for the non-linear relationship between air pollutant concentrations and their sources of emission and dispersion [11]. A general class of statistical models such as analysis of covariance (ANCOVA) models have been proven to be effective for analyzing observational data because it considers the confounding effects and complex interactions among the variables. The ANCOVA model provides relatively clean estimates of the association between explanatory X variables (quantitative and qualitative) and dependent Y variable/outcome (quantitative) [12]. The ANCOVA method has been used to examine the association between long-term exposure to atmospheric PM and neurocognitive outcomes and brain volumes of older women in the United States [13]. However, the study found no evidence of increased risks of mild cognitive impairment and dementia associated with long-term PM exposure.

One of the most popular ML models that can be used to solve regression and classification problems is the random forest model. Compared to other ML models (such as neural networks and support vector machines), the advantages of the random forest model are (1) high estimation accuracy, (2) unlikely to overfit, (3) easy data preparation (ability to handle missing values and no requirements for normalization and scaling), (4) able to handle non-linearity and high-order interactions between explanatory variables, and (5) sophisticated output with variable importance [14,15]. The main disadvantage of the random forest model is that it can be slow in estimation when the number of trees is large although the model is more robust [16,17]. Random forest models have been applied to estimate air pollutant concentrations in various countries. For example, a random forest model has been applied to estimate daily PM_2.5_, PM_2.5–10_ (2.5 µm ≤ PM ≤ 10 µm) and PM_10_ (PM ≤ 10 µm) concentrations from the year 2013 to 2015 (3 years) in Italy in 2019 [15]. Their models were able to capture about 75% to 80% of PM_2.5_ and PM_10_ variability. However, their model for PM_2.5–10_ performed poorly, and this was due to the limited availability of PM_2.5_ monitors and missing data. Another recent study has applied a random forest approach to estimate daily PM_2.5_, PM_2.5–10_, PM_10_, NO_2_ and O_3_ concentrations from 2005 to 2016 (12 years) in Sweden in 2020 [18]. Although their models were able to describe the variability of 69% for PM_2.5_, 65% for PM_2.5–10_, 64% for PM_10_, 74% for NO_2_ and 78% for O_3_, there was high collinearity among several covariates being added as predictors/explanatory variables in their model.

Meteorological parameters such as wind speed, wind direction, rainfall and air temperature are known to influence air pollutant dispersion and they can be included in the model as explanatory variables. In 2017, a classification model for estimating PM_2.5_ concentration between June 2007 and July 2013 (6 years) in Ecuador had been developed by the ML approach from daily meteorological data of wind speed, wind direction and rainfall [19]. That model could only estimate PM_2.5_ concentration up to 20 µg/m^3^. Additional parameters such as daily air temperature, solar radiation and air pressure might need to be included in the model to be able to estimate PM_2.5_ concentration above 20 µg/m^3^. In 2015, a study was conducted to estimate the NO_2_ concentration in Romania from 2009 to 2013 (5 years) by multiple linear regression (MLR) and artificial neural networks (ANNs), focusing on the dependence between meteorological parameters (such as air temperature, air pressure, wind speed, wind direction, solar radiation, rainfall and relative humidity) and their influence on measured NO_2_ concentration [20]. Their results show that meteorological parameters have an impact on the NO_2_ concentration although their estimation models have relatively low accuracy, which could have resulted from the measurement errors of the meteorological parameters.

Many studies have developed models to estimate and/or predict air pollutant concentrations within a country but the use of those models to another country of interest has been scarcely explored and it has been a challenge because of the lack of air quality, emission inventory and meteorological data from different countries. Nevertheless, cross-country models, when developed, can enhance the capability of cross-border surveillance of air quality with cost-effective monitoring plans. Hence, the present study aimed to develop and evaluate ML-based models that could estimate daily PM_2.5_ and PM_10_ concentrations in Brunei Darussalam from January 2009 to December 2019 (11 years) using models developed from air quality and meteorological data in Singapore from March 2016 to February 2018 (2 years) and meteorological data from Brunei Darussalam. The performance of the estimation models was evaluated for overall air quality as well as for good and moderate air quality at different seasons against observed PM concentrations in each country. Although the duration of available data sets in individual countries varies significantly involving respective influences exerted by changes in social and economic patterns, this study intends to demonstrate that employing selected variables through ML is possible to develop correlations for inter-country estimations despite insufficient information (for example, anthropogenic and natural emissions data). The study has three objectives, which are:

1. To generate ML-based ANCOVA and random forest regression (RFR) models from Singapore’s air quality and meteorological data for estimating daily PM_2.5_ and PM_10_ concentrations in Singapore and to assess the models’ estimation performance;
2. To determine the most important explanatory variable that influenced the model outcome;
3. To apply and assess the performance of the derived models for estimating daily PM_2.5_ and PM_10_ concentrations in Brunei Darussalam.

## 2. Materials and Methods

### 2.1. Study Areas

The study will examine the concentrations of PM in two Southeast Asian countries, namely Brunei Darussalam (4.5353° N, 114.7277° E) and Singapore (1.3521° N, 103.8198° E), as examples. These two countries often encounter transboundary smoke haze events. Brunei Darussalam has a population of 453,600 (as of June 2020) [21] with a total land area of 5765 square kilometers [22] and Singapore has a population of 5.69 million (as of June 2020) [23] with a total land area of 728 square kilometers (as of June 2020) [24]. Both countries have a tropical equatorial climate with warm and uniform air temperatures (mean daily air temperature range: 18 °C to 38 °C in Brunei Darussalam and 24 °C to 32 °C in Singapore), low wind speed (mean wind speed: <0.5 m/s in Brunei Darussalam and <2.5 m/s in Singapore), winds mostly blowing from the south direction in Brunei Darussalam and from the northeast and the south direction in Singapore, and heavy rainfall (mean annual rainfall total: >2300 mm in Brunei Darussalam and >2100 mm in Singapore) over the year [25,26]. These meteorological parameters do not show large monthly variations, but they show prominent daily variations due to the strong relation with solar heating [26].

**Figure 1:**
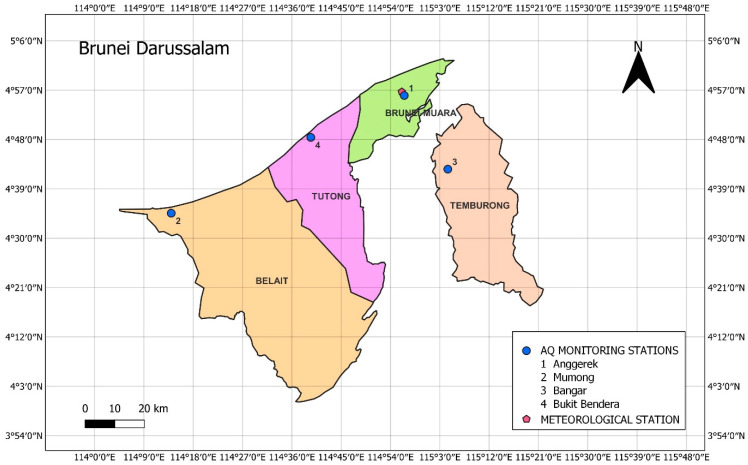
Locations of 4 air quality (AQ) monitoring stations across the four districts and a meteorological station in Brunei-Muara district in Brunei Darussalam.

**Figure 2:**
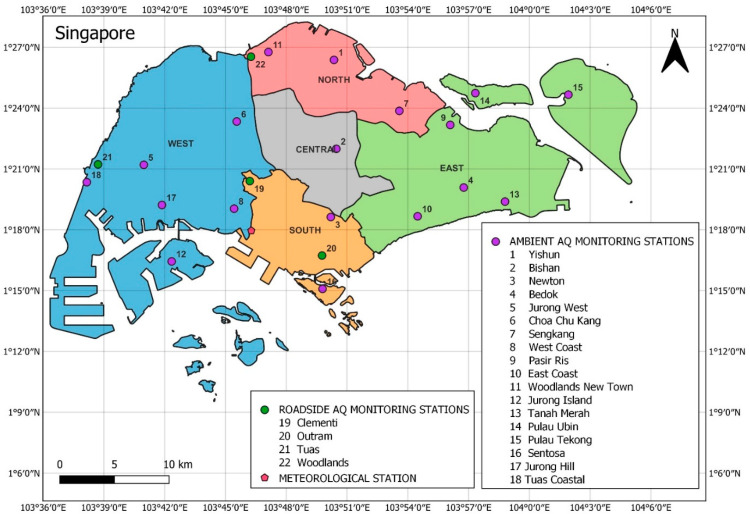
Locations of 22 air quality (AQ) monitoring stations (18 monitor general air quality and 4 monitor roadside air quality) across the five air quality regions and a meteorological station in the southern region in Singapore.

The air quality in the four districts (i.e., Belait, Tutong, Brunei-Muara and Temburong) of Brunei Darussalam was assessed through 4 air quality monitoring stations (1 in each district) and the meteorological monitoring station is located at the Brunei International Airport in Brunei-Muara district (Figure 1). In Singapore, there are 22 air quality monitoring stations (18 monitor general ambient air quality and 4 monitor roadside air quality) installed in different parts across its five air quality regions (north, south, east, west and central) [27], and the meteorological monitoring station is located at the rooftop of the Faculty of Engineering’s building of the National University Singapore (NUS) (Figure 2) [28].

### 2.2. Data Collection and Preparation

To develop PM estimation models, air quality and meteorological data from Singapore between March 2016 and February 2018 (2 years) were collected. The air quality monitoring data were from five regions (north, south, east, west and central) in Singapore and they were downloaded from online data provided by the National Environment Agency (NEA) of Singapore (https://www.haze.gov.sg/resources/pollutant-concentrations; accessed on 15 January 2020). The meteorological data were observed from the National University Singapore (NUS) weather station, and they were obtained from a weather portal (https://www.nusurbanclimate.com/weather-portal; accessed on 17 February 2020, courtesy of Professor Matthias Roth, Department of Geography, NUS). The collected air quality monitoring data from Singapore comprises of 1-h average hourly PM_2.5_ concentration (µg/m^3^), 24-h average hourly PM_10_ concentration (µg/m^3^) and air quality condition (good, moderate or unhealthy), and the corresponding meteorological data comprise of hourly measurements of air temperature (°C), wind speed (m/s), wind direction (°) and rainfall (mm). Air quality is considered in good condition when the Pollutant Standards Index (PSI) value is between 0 and 50, moderate condition when the PSI is between 51 and 100 and unhealthy condition when the PSI is between 101 and 200 [29].

To test the applicability of the derived PM estimation models to another country in the same region, air quality and meteorological data from Brunei Darussalam between January 2009 and December 2019 (11 years) were collected. The daily air quality monitoring data were from four districts (Belait, Tutong, Brunei-Muara and Temburong) in Brunei Darussalam and they were provided by the Department of Environment, Parks and Recreation (JASTRe). The meteorological data were observed from a meteorological station in Brunei-Muara district, and they were provided by the Brunei Darussalam Meteorological Department (BDMD). The collected air quality monitoring data from Brunei Darussalam include daily average PM_10_ concentration (µg/m^3^) and air quality condition (good, moderate or unhealthy), and its meteorological data include daily average measurements of air temperature (°C), wind speed (m/s), wind direction (°) and rainfall (mm). Based on the approach described by the WHO Air Quality Guidelines [30], the (unavailable) daily average PM_2.5_ concentration (known as ‘theoretical’ PM_2.5_ concentration in this study) in Brunei Darussalam was estimated by multiplying the daily average PM_10_ concentration with the average factor of PM_2.5_ over PM_10_ (PM_2.5_/PM_10_) in Brunei Darussalam, which was 0.43. The value of this average factor of PM_2.5_/PM_10_ was considered close to the typical value of 0.5 for developing country urban areas stated in the WHO Air Quality Guidelines [30] and it was determined from the PM_2.5_ concentration data reported by the Organisation for Economic Co-operation and Development (OECD) [31] and the corresponding PM_10_ concentration data provided by JASTRe between 2010 and 2019 for Brunei Darussalam. 

Time parameters such as day, month, year and monsoon season were also considered as variables affecting the PM concentration in the region. The monsoon seasons of both countries are north-east (NE) monsoon (from December to March), Inter-monsoon 1 (from April to May), south-west (SW) monsoon (from June to September) and Inter-monsoon 2 (from October to November) [26]. The collected data were grouped into 716 daily average observations of PM concentrations from five regions in Singapore and 4015 daily average observations of PM concentrations from four districts in Brunei Darussalam with their corresponding air quality condition, meteorological data and monsoon season for analysis using XLSTAT software. The data used in this study were restricted to observations during good and moderate conditions due to the limited availability of data during unhealthy air quality conditions in both countries. The models’ inputs for both countries consist of 10 explanatory variables, in which 8 of the variables were quantitative in nature and 2 of the variables were qualitative in nature. For PM_2.5_ concentration estimation, the models’ inputs were day, month, year, monsoon season, daily PM_10_ concentration, air temperature, wind speed, wind direction, rainfall and air quality condition. For PM_10_ concentration estimation, the models’ inputs were day, month, year, monsoon season, daily PM_2.5_ concentration, air temperature, wind speed, wind direction, rainfall and air quality condition.

### 2.3. Machine Learning (ML) Techniques

Based on the nature of the dependent variable Y to estimate and the nature of the explanatory X variables, two regression models were explored, namely: (i) analysis of covariance (ANCOVA) and (ii) random forest regression (RFR). The data were randomly separated into two samples in which 80% of the observations were used for model learning/training and 20% of the remaining observations were used for model validation [32]. First, these two models were trained, validated and tested with air quality and meteorological data in Singapore. Then, these derived models for estimating daily PM_2.5_ and PM_10_ concentrations were applied to new observations with data from Brunei Darussalam. The performance of the ANCOVA and RFR models in estimating daily PM_2.5_ and PM_10_ concentrations for overall air quality as well as for good and moderate air quality conditions during different monsoon seasons in both countries was evaluated based on statistical indicators such as the determination coefficient ($R^{2}$) and root mean square of the error (RMSE).

#### 2.3.1. Analysis of Covariance (ANCOVA) Model

ANCOVA analysis was implemented, considering the interactions between the quantitative and qualitative explanatory variables. The interaction between explanatory variables A and B (also known as an interaction variable) was represented by the notation “*A* × *B*”, which is the product of the explanatory variables A and B [33]. The maximum interaction level of the model was 2. The stepwise variables selection method with an entry probability of 0.05 and a removal probability of 0.10 was chosen for the model. A multiple comparison test was applied to all factors (qualitative variables including the interactions between qualitative variables) to determine if the parameters for the various qualitative variables of a factor differ significantly or not. The comparisons were made between all pairs of qualitative variables with a control variable based on the mean squared error (MSE) that was associated with an interaction term in the model [32].

#### 2.3.2. Random Forest Regression (RFR) Model

Estimation models for daily PM_2.5_ and PM_10_ concentrations can also be developed by the RFR method with bootstrap aggregating (known as bagging). This method aggregates a group of explanatory variables in the form of classification and regression trees (CART) from different bootstrap samples to obtain a more efficient final explanatory variable. The forest sampling method used in this study was random with replacement. The desired number of trees in the forest was 100 and the depth of the maximum tree was 20. The performance of the RFR model was evaluated by the MSE of the validation sample. The importance of a given variable was measured by the mean increase error (MIE) of a tree when the observed values of this variable were randomly exchanged in the out-of-bag (OOB) samples (i.e., data that were not included in the bootstrap samples at each iteration of the forest). The higher the MIE value, the greater the importance of the variable for the model would be [32].

## 3. Results and Discussion

### 3.1. Data Summary

The descriptive statistics of the measured quantitative variables (i.e., PM_2.5_ and PM_10_ concentrations, air temperature, wind speed, wind direction and rainfall) from all monitoring areas in Singapore and Brunei Darussalam are summarized in Table 1. The overall mean daily PM concentrations in Singapore from March 2016 to February 2018 were 14.46 µg/m^3^ for PM_2.5_ and 26 µg/m^3^ for PM_10_, and those in Brunei Darussalam from January 2009 to December 2019 were 7.67 µg/m^3^ for PM_2.5_ and 18.02 µg/m^3^ for PM_10_. The overall mean daily concentrations of PM in these two countries were within the WHO air quality guideline limits for PM, in which the 24-h mean guideline values are 25 µg/m^3^ for PM_2.5_ and 50 µg/m^3^ for PM_10_ [30]. No large daily mean variation was observed for air temperature, wind speed and rainfall in these two countries. Due to different geographical locations, the prevailing winds were from the south-southeast direction (15% of the observations) in Singapore and from the north-northeast direction (96% of the observations) in Brunei Darussalam. In the selected time periods, the overall air quality in Singapore was frequently in moderate condition (52% of the observations) and good condition (48% of the observations), whereas the overall air quality in Brunei Darussalam was often in good condition (99% of the observations) and occasionally in moderate condition (1% of the observations).

**Figure 3:**
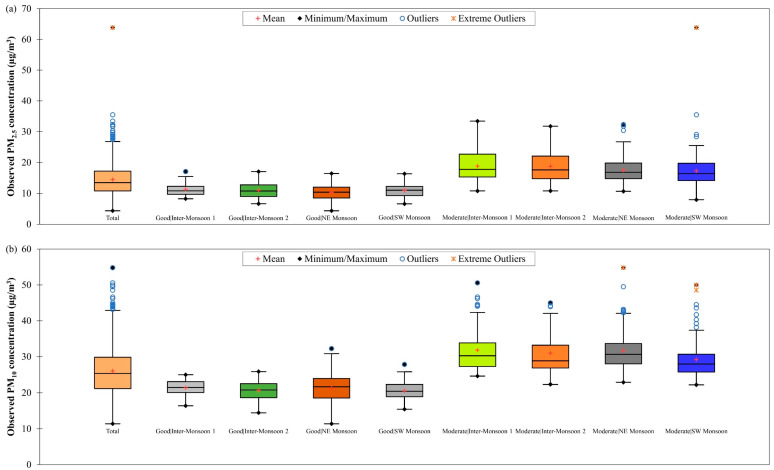
Box plots of observed daily (**a**) PM_2.5_ and (**b**) PM_10_ concentrations in Singapore from March 2016 to February 2018 during good and moderate air quality conditions in different monsoon seasons.

Figure 3 shows the box plots of observed PM_2.5_ and PM_10_ concentrations in Singapore from March 2016 to February 2018 during good and moderate air quality conditions in different monsoon seasons. Daily PM_2.5_ concentration in Singapore was ranged from 4.38 µg/m^3^ to 17.06 µg/m^3^ during good air quality condition and from 7.95 µg/m^3^ to 63.84 µg/m^3^ during moderate air quality condition. The highest daily PM_2.5_ concentration was found to be 63.84 µg/m^3^ and it was observed during SW monsoon season. This was mainly attributed by smoke haze from large-scale forest and peatland biomass burning in Sumatra and Kalimantan (islands in Indonesia) that had been blown by the prevailing southwest winds towards Singapore [34]. As for the daily PM_10_ concentration in Singapore, it was ranged from 11.38 µg/m^3^ to 32.25 µg/m^3^ during good air quality condition and from 22.18 µg/m^3^ to 54.80 µg/m^3^ during moderate air quality condition. The highest daily PM_10_ concentration was found to be 54.80 µg/m^3^ and it was observed during NE monsoon season mainly due to forest, shrubland and grassland biomass burning in Mainland Southeast Asia [35,36,37].

The PM_10_ concentration was not recorded at the highest concentration and the value was lower than PM_2.5_ during the SW monsoon season because of the difference in types of biomasses burnt [38] during SW and NE monsoon seasons in the region. Generally, PM_2.5_ emissions were higher during forest and peatland biomass burning while PM_10_ emissions were higher during shrubland, crop residual and grassland biomass burning [38,39]. There were several outliers (values that fall outside 1.5 times the interquartile range (IQR) of the third quartile (Q3); Q3 + 1.5IQR) and extreme outliers (values that fall outside 3 times the IQR of the Q3; Q3 + 3IQR) seen in Figure 3. This indicates that Singapore experienced high and extreme particulate events that led to increased PM_2.5_ and PM_10_ concentrations.

**Figure 4:**
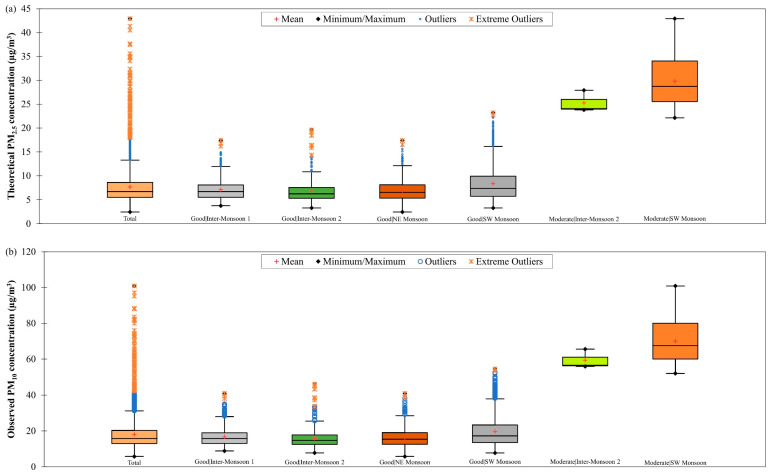
Box plots of (**a**) theoretical daily PM_2.5_ concentration and (**b**) observed daily PM_10_ concentration in Brunei Darussalam from January 2009 to December 2019 during good and moderate air quality conditions in different monsoon seasons.

The box plots of theoretical PM_2.5_ and observed PM_10_ concentrations in Brunei Darussalam from January 2009 to December 2019 during good and moderate air quality conditions in different monsoon seasons are shown in Figure 4. In Brunei Darussalam, the range of theoretical daily PM_2.5_ concentration was from 2.45 µg/m^3^ to 23.19 µg/m^3^ during good air quality conditions and from 22.13 µg/m^3^ to 42.93 µg/m^3^ during moderate air quality conditions. The highest theoretical daily PM_2.5_ concentration was expected during SW monsoon season with a value of 42.93 µg/m^3^ as a result of transboundary smoke haze events caused by biomass burning in the region. The range of observed PM_10_ concentration in Brunei Darussalam was from 5.75 µg/m^3^ to 54.50 µg/m^3^ during good air quality conditions and from 52 µg/m^3^ to 100.90 µg/m^3^ during moderate air quality conditions. The highest daily PM_10_ concentration was found to be 100.90 µg/m^3^ and it was observed during SW monsoon season due to smoke haze from hotspots in the Borneo region that had been blown by the prevailing southwest winds to Brunei Darussalam [40]. Numerous outliers and extreme outliers were seen in Figure 4, indicating that Brunei Darussalam also experienced high and extreme particulate events that contributed to the increase in the concentrations of PM.

### 3.2. Estimation Models for PM_2.5_ Concentration

**Figure 5:**
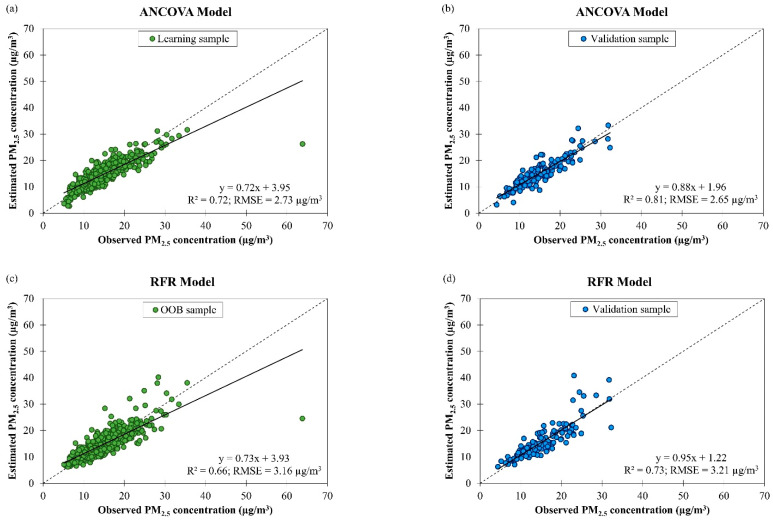
Scatter plots of estimated daily PM_2.5_ concentration against observed daily PM_2.5_ concentration in Singapore from March 2016 to February 2018 by (**a**,**b**) ANCOVA and (**c**,**d**) RFR models with learning and validation samples.

Figure 5 shows the scatter plots of estimated daily PM_2.5_ concentration against observed daily PM_2.5_ concentration by ANCOVA and RFR models based on air quality and meteorological data in Singapore from March 2016 to February 2018 with learning and validation samples. Evaluation of both models’ performances in model learning and validation showed that the ANCOVA model produced better fitting and accuracy ($R^{2}$ = 0.72 and RMSE = 2.73 µg/m^3^ for model learning; $R^{2}$ = 0.81 and RMSE = 2.65 µg/m^3^ for model validation) than the RFR model ($R^{2}$ = 0.66 and RMSE = 3.16 µg/m^3^ for model learning; $R^{2}$ = 0.73 and RMSE = 3.21 µg/m^3^ for model validation) in estimating daily PM_2.5_ concentration in Singapore. Eight variables (i.e., (day × air quality condition), (year × air quality condition), (PM_10_ concentration × air quality condition), (year × wind speed), (air temperature × wind speed), (air temperature × wind direction), (air temperature × PM_10_ concentration), (wind direction × PM_10_ concentration)) were retained in the ANCOVA model when the stepwise variables selection method was employed.

Based on the sum of squares of the errors (SSE) analysis on the ANCOVA model (refer Table 2), variables (day × air quality condition), (year × air quality condition) and (PM_10_ concentration × air quality condition) bring significant information to explain the variability of the dependent variable PM_2.5_ concentration. The most influential variable among the explanatory variables was the interaction variable (PM_10_ concentration ∗ air quality condition) because it has the highest MSE value (171.98 µg/m^3^) with a relatively low probability associated with the F value (2 × $10^{-6}$) when this variable was removed from the ANCOVA model (Table 2). This can be explained by the fact that PM_2.5_ is a subset of PM_10_ and that PM_10_ is one of the determining factors of air quality condition.

The equation of the best ANCOVA model for estimating daily PM_2.5_ concentration (µg/m^3^) with significant explanatory variables is provided in Equation (1):

(1) $${\mathrm{PM}}_{2.5}=6.11+\left[\left(4.79\times 10^{-2}\right)\left(D\times AQ_{Moderate}\right)\right]-\left[\left(3.65\times 10^{-3}\right)\left(Y\times AQ_{Moderate}\right)\right]+0.29\left({\mathrm{PM}}_{10}\times AQ_{Moderate}\right)$$

where D is the day, Y is the year, ${\mathrm{PM}}_{10}$ represents the observed daily PM_10_ concentration (µg/m^3^) and $AQ_{Moderate}$ represents moderate air quality condition. Equation (1) indicates that daily PM_2.5_ concentration could be estimated if the corresponding day, year and PM_10_ concentration during moderate air quality were available. The order of variable importance based on MIE for estimating daily PM_2.5_ concentration by RFR model was (from high to low): PM_10_ (MIE = 28.05 µg/m^3^), air quality condition (MIE = 3.64 µg/m^3^), rainfall (MIE = 1.89 µg/m^3^), air temperature (MIE = 1.11 µg/m^3^), wind direction (MIE = 0.62 µg/m^3^), month (MIE = 0.32 µg/m^3^), day (MIE = 0.01 µg/m^3^), wind speed (MIE = −0.50 µg/m^3^), year (MIE = −0.62 µg/m^3^) and monsoon season (MIE = −1.18 µg/m^3^). This shows that the most important variable for estimating daily PM_2.5_ concentration in Singapore by the RFR model was PM_10_ concentration.

**Figure 6:**
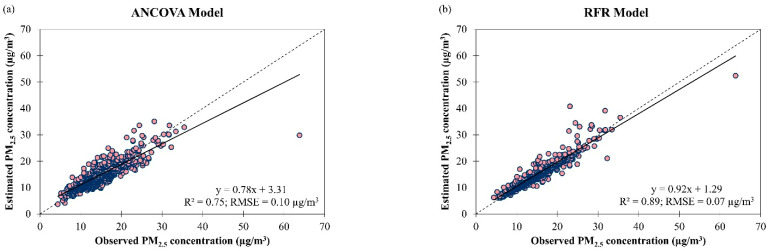
Scatter plots of estimated daily PM_2.5_ concentration against observed daily PM_2.5_ concentration by (**a**) ANCOVA and (**b**) RFR models for overall air quality in Singapore from March 2016 to February 2018.

When the ANCOVA and RFR models were tested using all the observational data in Singapore from March 2016 to February 2018, both models yielded higher accuracy (in terms of RMSE) compared to when they were trained and validated with the corresponding datasets. During model testing, the ANCOVA model showed poorer fitting and accuracy ($R^{2}$ = 0.75 and RMSE = 0.10 µg/m^3^) than the RFR model ($R^{2}$ = 0.89 and RMSE = 0.07 µg/m^3^) in estimating daily PM_2.5_ concentration in Singapore, in general (Figure 6). Due to these reasons, the difference between the estimated and observed daily PM_2.5_ concentrations in Singapore was larger for the ANCOVA model (underestimation by 11% on 48% of the observations and overestimation by 15% on 52% of the observations) compared to the RFR model (underestimation by 6% on 47% of the observations and overestimation by 9% on 53% of the observations). The best estimation (i.e., the intersection point between the best fit line/trendline and the diagonal (y = x) line) of PM_2.5_ concentration in Singapore was at 15.05 µg/m^3^ for the ANCOVA model and 15.75 µg/m^3^ for the RFR model. Besides the meteorological parameters, other air pollutants (such as carbon monoxide (CO) and nitrogen oxides (NO_x_)) concentration data could be added to the model in the future as explanatory variables to further improve the model performance for estimating daily PM_2.5_ concentration since they were found to be associated with PM_2.5_ concentrations in São Paulo, Brazil for CO [41] and in Fresno, California, USA for NO_x_ [42].

**Figure 7:**
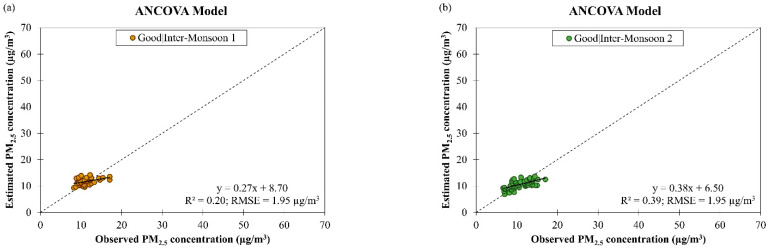

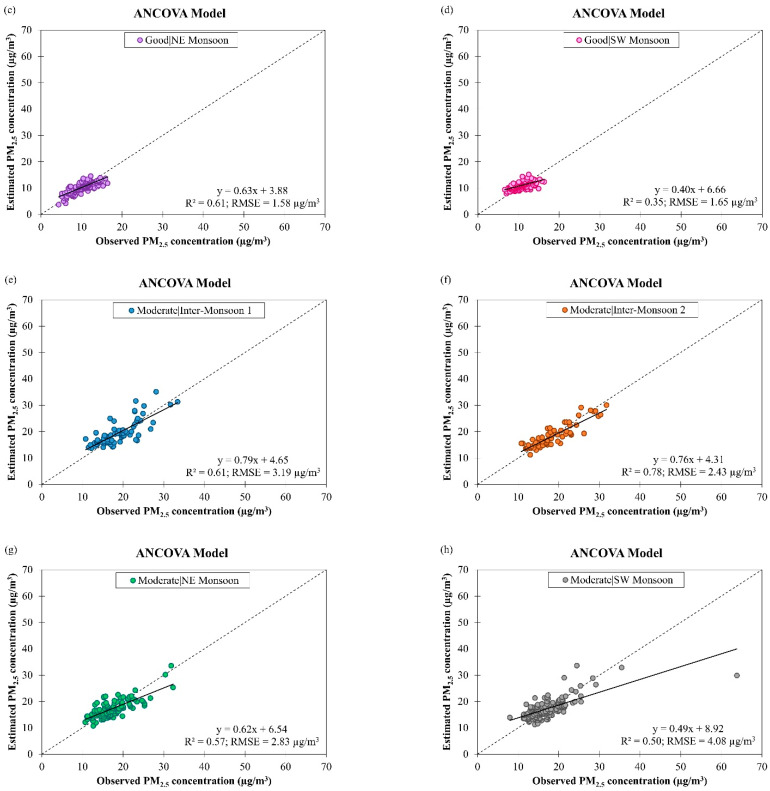
Scatter plots of estimated daily PM_2.5_ concentration against observed daily PM_2.5_ concentration by ANCOVA model for (**a**–**d**) good and (**e**–**h**) moderate air quality conditions during different monsoon seasons in Singapore from March 2016 to February 2018.

The models’ performances during good and moderate air quality conditions in different monsoon seasons in Singapore from March 2016 to February 2018 were also evaluated. Figure 7 showed that the accuracy of the ANCOVA model was reduced (as indicated by an increment in the RMSE value from 1.78 µg/m^3^ to 3.13 µg/m^3^, on average) when the air quality condition changed from good to moderate despite having better data fitting (as indicated by an increment in the $R^{2}$ value from 0.39 to 0.62, on average). This implies that the ANCOVA model may not be able to handle increased PM_2.5_ concentration well. The $R^{2}$ value of the ANCOVA model for estimating daily PM_2.5_ concentration in different monsoon seasons in Singapore was ranged between 0.20 and 0.61 during good air quality and between 0.50 and 0.78 during moderate air quality (Table 3). The RMSE value of the ANCOVA model for estimating daily PM_2.5_ concentration in different monsoon seasons in Singapore was ranged between 1.58 µg/m^3^ and 1.95 µg/m^3^ during good air quality and between 2.43 µg/m^3^ and 4.08 µg/m^3^ during moderate air quality (Table 3). The highest RMSE value (4.08 µg/m^3^) was attained during SW monsoon season when the air quality was moderate, and this was because of the large variation in daily PM_2.5_ concentration (as indicated by the outliers and extreme outliers in Figure 3a), as a result of the smoke haze event that often occurs in this season. The ANCOVA model exhibited the best performance for daily PM_2.5_ concentration estimation during NE monsoon season when the air quality was good with $R^{2}$ value of 0.61 and RMSE value of 1.58 µg/m^3^.

**Figure 8:**
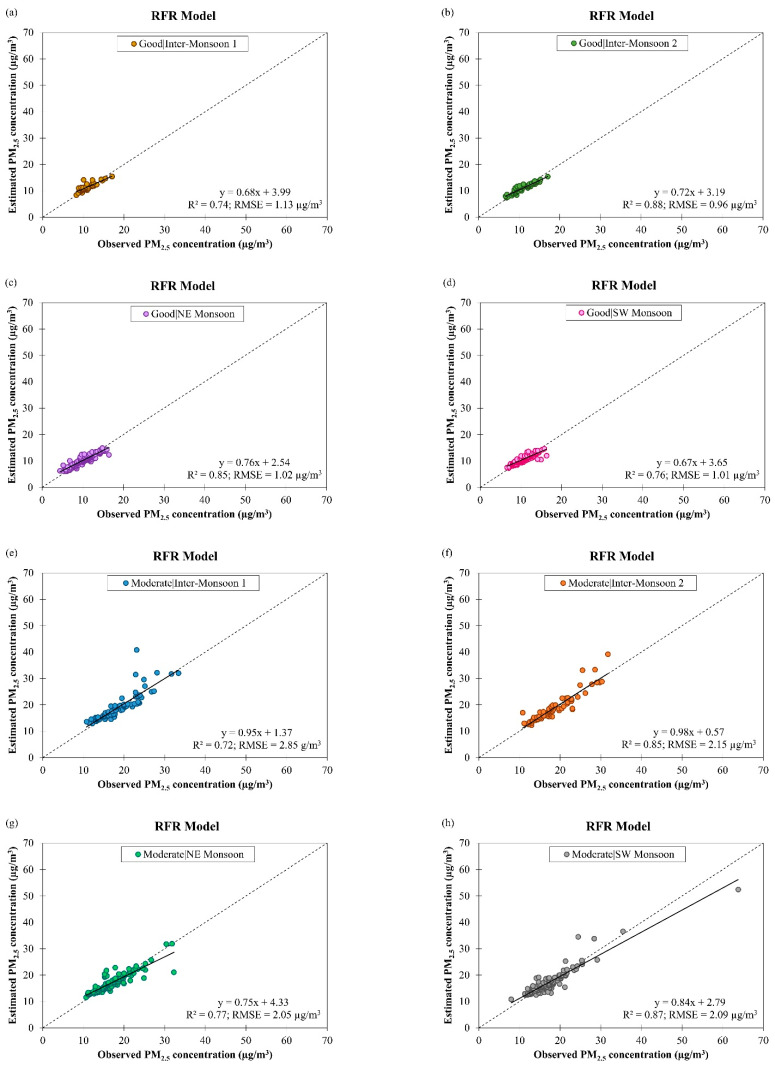
Scatter plots of estimated daily PM_2.5_ concentration against observed daily PM_2.5_ concentration by RFR model for (**a**–**d**) good and (**e**–**h**) moderate air quality conditions during different monsoon seasons in Singapore from March 2016 to February 2018.

Figure 8 shows the scatter plots of observed and estimated daily PM_2.5_ concentrations by the RFR model for good and moderate air quality conditions during different monsoon seasons in Singapore from March 2016 to February 2018. It can be seen that the accuracy of the RFR model was reduced (as indicated by an increment in the RMSE value from 1.03 µg/m^3^ to 2.29 µg/m^3^, on average) and the data fitting was slightly affected (as indicated by a very small decrement in the $R^{2}$ value from 0.81 to 0.80, on average) when the air quality condition changed from good to moderate. This implies that the RFR model may have a limitation in handling increased PM_2.5_ concentration. The ranges of $R^{2}$ value of the RFR model for estimating daily PM_2.5_ concentration in different monsoon seasons in Singapore were between 0.74 and 0.88 during good air quality and between 0.72 and 0.87 during moderate air quality. The ranges of RMSE value of the RFR model for estimating daily PM_2.5_ concentration in different monsoon seasons in Singapore was between 0.96 µg/m^3^ and 1.13 µg/m^3^ during good air quality and between 2.05 µg/m^3^ and 2.85 µg/m^3^ during moderate air quality. Comparison of the ANCOVA and RFR models’ performance for daily PM_2.5_ concentration estimation during good and moderate air quality in different monsoon seasons in Singapore showed that the RFR model was more accurate with better data fitting ($R^{2}$ = 0.81 and RMSE = 1.66 µg/m^3^, on average) than the ANCOVA model ($R^{2}$ = 0.50 and RMSE = 2.46 µg/m^3^, on average) (Table 3).

### 3.3. Estimation Models for PM_10_ Concentration

**Figure 9:**
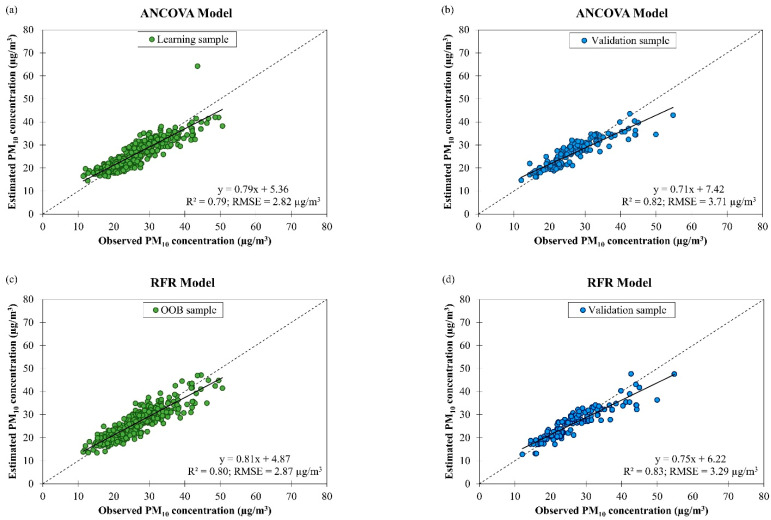
Scatter plots of estimated daily PM_10_ concentration against observed daily PM_10_ concentration in Singapore from March 2016 to February 2018 by (**a**,**b**) ANCOVA and (**c**,**d**) RFR models with learning and validation samples.

Scatter plots of estimated daily PM_10_ concentration against observed daily PM_10_ concentration by ANCOVA and RFR models based on air quality and meteorological data in Singapore from March 2016 to February 2018 with learning and validation samples are illustrated in Figure 9. Both models’ performances in model learning and validation were evaluated and the results show that the ANCOVA model has comparable data fitting and accuracy ($R^{2}$ = 0.79 and RMSE = 2.82 µg/m^3^) to the RFR model ($R^{2}$ = 0.80 and RMSE = 2.87 µg/m^3^) in model learning and the RFR model has comparable data fitting and accuracy ($R^{2}$ = 0.83 and RMSE = 3.29 µg/m^3^) to the ANCOVA model ($R^{2}$ = 0.82 and RMSE = 3.71 µg/m^3^) in model validation. Using the stepwise variables selection method, ten variables (i.e., (year × wind speed), (year × PM_2.5_ concentration), (air temperature × PM_2.5_ concentration), (wind speed × PM_2.5_ concentration), (wind speed × air quality condition), (wind direction × PM_2.5_ concentration), (wind direction × air quality condition), (PM_2.5_ concentration × monsoon season), (year × air temperature), and (air temperature × wind speed)) were retained in the ANCOVA model.

Results of the sum of squares of the errors (SSE) analysis on the ANCOVA model (Table 4), indicates that variables (year × wind speed), (year × PM_2.5_ concentration), (air temperature × PM_2.5_ concentration), (wind speed × PM_2.5_ concentration), (wind speed × air quality condition), (wind direction × PM_2.5_ concentration), (wind direction × air quality condition) and (PM_2.5_ concentration × monsoon season) bring significant information to explain the variability of the dependent variable PM_10_ concentration. Among the explanatory variables, the interaction variable (wind direction × air quality condition) was the most influential because it has the lowest probability associated with the F value (3.58 ×$10^{-6}$), highest SSE value (204.49 µg/m^3^) and relatively high MSE value (102.25 µg/m^3^) when this variable was removed from the ANCOVA model (see Table 4). This could be due to the effect of wind direction on PM_10_ concentration in the area [43] and the fact that PM_10_ is one of the determining factors of air quality condition.

The equation of the best ANCOVA model for estimating daily PM_10_ concentration (µg/m^3^) with significant explanatory variables is provided in Equation (2):

(2) $$\begin{array}{cc} {\mathrm{PM}}_{10}=-39.43 & +\left[\left(8.68\times 10^{-4}\right)\left(Y\times \;T\right)\right]+\left[\left(5.10\times 10^{-3}\right)\left(Y\times \;WS\right)\right]+\left[\left(1.85\times 10^{-3}\right)\left(Y\times {\;\mathrm{PM}}_{2.5}\right)\right]-0.31\left(T\times \;WS\right)\\ & -\left[\left(9.24\times 10^{-2}\right)\left(T\times {\;\mathrm{PM}}_{2.5}\right)\right]-\left[\left(7.25\times 10^{-2}\right)\left(WS{\;\mathrm{PM}}_{2.5}\right)\right]+0.84\left(WS\times \;AQ_{Moderate}\right)\\ & -\left[\left(1.15\times 10^{-3}\right)\left(WD\times {\;\mathrm{PM}}_{2.5}\right)\right]+\left[\left(1.44\times 10^{-2}\right)\left(WD\times \;AQ_{Moderate}\right)\right]-0.05\left({\mathrm{PM}}_{2.5}\times \times M_{IM2}\right)\\ & -\left[\left(8.45\times 10^{-2}\right)\left({\mathrm{PM}}_{2.5}\times \;M_{\mathrm{NE}}\right)\right]-\left[\left(7.41\times 10^{-2}\right)\left({\mathrm{PM}}_{2.5}\;\times M_{\mathrm{SW}}\right)\right] \end{array}$$

where Y is the year, T is the air temperature (°C), WS is the wind speed (m/s), WD is the wind direction (°), ${\mathrm{PM}}_{2.5}$ represents the observed daily PM_2.5_ concentration (µg/m^3^), $AQ_{Moderate}$ represents moderate air quality condition, $M_{IM2}$ is the inter-monsoon 2 season, $M_{\mathrm{NE}}$ is the NE monsoon season and $M_{\mathrm{SW}}$ is the SW monsoon season. Equation (2) indicates that the daily PM_10_ concentration could be estimated if the corresponding year, temperature, wind speed, wind direction, PM_2.5_ concentration during moderate air quality in NE, SW and inter-monsoon 2 seasons were available. The order of variable importance based on MIE for estimating daily PM_10_ concentration by RFR model was (from high to low): air quality condition (MIE = 51.35 µg/m^3^), PM_2.5_ (MIE = 46.78 µg/m^3^), wind direction (MIE = 11.59 µg/m^3^), wind speed (MIE = 7.01 µg/m^3^), air temperature (MIE = 4.32 µg/m^3^), year (MIE = 3.72 µg/m^3^), month (MIE = 3.35 µg/m^3^), day (MIE = 2.99 µg/m^3^), rainfall (MIE = 1.45 µg/m^3^) and monsoon season (MIE = −2.68 µg/m^3^). This shows that air quality condition was the most important variable of RFR model for daily PM_10_ concentration estimation in Singapore.

**Figure 10:**
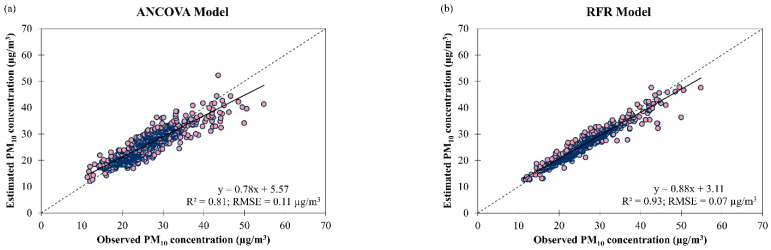
Scatter plots of estimated daily PM_10_ concentration against observed daily PM_10_ concentration by (**a**) ANCOVA and (**b**) RFR models for overall air quality in Singapore from March 2016 to February 2018.

The ANCOVA and RFR models for daily PM_10_ concentration estimation were tested on all the observational data in Singapore from March 2016 to February 2018 and the results show improvement in accuracy (in terms of RMSE) for both models and data fitting, particularly for the RFR model, compared to model learning and validation. The best model for overall estimation of PM_10_ concentration in Singapore was the RFR model as it showed better data fitting and higher accuracy ($R^{2}$ = 0.93 and RMSE = 0.07 µg/m^3^) than the ANCOVA model ($R^{2}$ = 0.81 and RMSE = 0.11 µg/m^3^) (Figure 10). It was found that the ANCOVA model underestimated daily PM_10_ concentration in Singapore by 8% (48% of the observations) and overestimated by 9% (52% of the observations) whereas the RFR model underestimated daily PM_10_ concentration in Singapore by 4% (46% of the observations) and overestimated by 5% (54% of the observations). The best estimation of PM_10_ concentration in Singapore was at 25.32 µg/m^3^ for the ANCOVA model and 25.92 µg/m^3^ for the RFR model (Figure 10). Both ANCOVA and RFR models developed in this study for daily PM_10_ concentration estimation outperformed the multiple non-linear regression (MNLR) model ($R^{2}$ = 0.36 and RMSE = 20.30 µg/m^3^, on average) for estimating daily PM_10_ concentration in three cities (Budapest, Miskolc and Pécs) in Hungary [44]. This could be due to more explanatory variables being used in the model development of this study compared to their study, which only has three explanatory variables (i.e., temperature, wind speed and boundary layer height).

**Figure 11:**
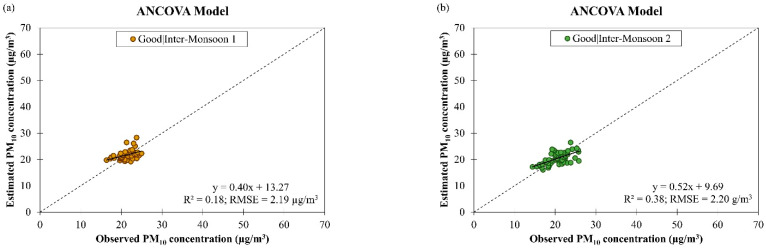

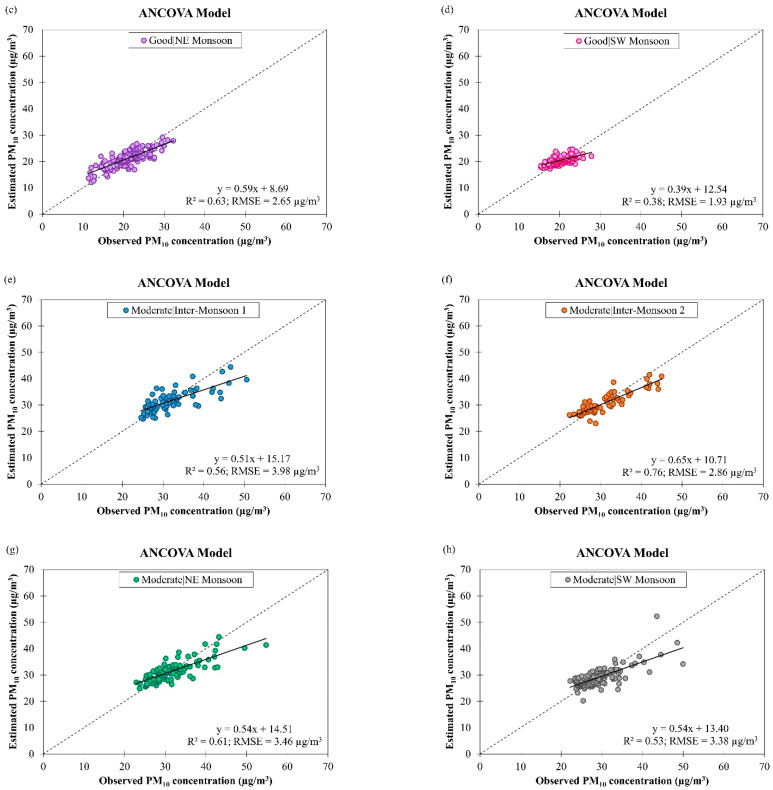
Scatter plots of estimated daily PM_10_ concentration against observed daily PM_10_ concentration by ANCOVA model for (**a**–**d**) good and (**e**–**h**) moderate air quality conditions during different monsoon seasons in Singapore from March 2016 to February 2018.

Figure 11 shows the observed and estimated daily PM_10_ concentrations by the ANCOVA model for good and moderate air quality conditions during different monsoon seasons in Singapore from March 2016 to February 2018. Although the model has better data fitting (as indicated by an increment in the $R^{2}$ value from 0.39 to 0.62, on average), its accuracy was reduced (as indicated by an increment in the RMSE value from 2.24 µg/m^3^ to 3.42 µg/m^3^, on average) when the good air quality condition became moderate. This shows that the ANCOVA model may not have good capability of handling increased PM_10_ concentration as well as PM_2.5_ concentration (as mentioned earlier in Section 3.2). A possible reason for this could be the low interaction level of the ANCOVA model used in this study, which may not be enough to account for the confounding effects between the input parameters of the model to describe the complex reality of atmospheric pollution. Therefore, it was suggested to use a higher interaction level for the ANCOVA model in future studies.

As shown in Table 3, the ANCOVA model for estimating daily PM_10_ concentration in different monsoon seasons in Singapore has $R^{2}$ value ranging between 0.18 and 0.63 during good air quality and between 0.53 and 0.76 during moderate air quality, and RMSE value ranging between 1.93 µg/m^3^ and 2.65 µg/m^3^ during good air quality and between 2.86 µg/m^3^ and 3.98 µg/m^3^ during moderate air quality. The highest RMSE value (3.98 µg/m^3^) was attained during moderate air quality in inter-monsoon 1 season, and this could be due to large variation in daily PM_10_ concentration (as indicated by the outliers in Figure 4b) as a result of the prolonged smoke haze event from the NE monsoon season, which limited the dispersion of PM_10_ by the meteorological parameters (for examples, wind speed, wind direction and rainfall) [45]. The ANCOVA model for daily PM_10_ concentration estimation in Singapore showed the best performance during good air quality in SW monsoon because it has the highest estimation accuracy with an RMSE value of 1.93 g/m^3^ although it has a low $R^{2}$ value of 0.38.

**Figure 12:**
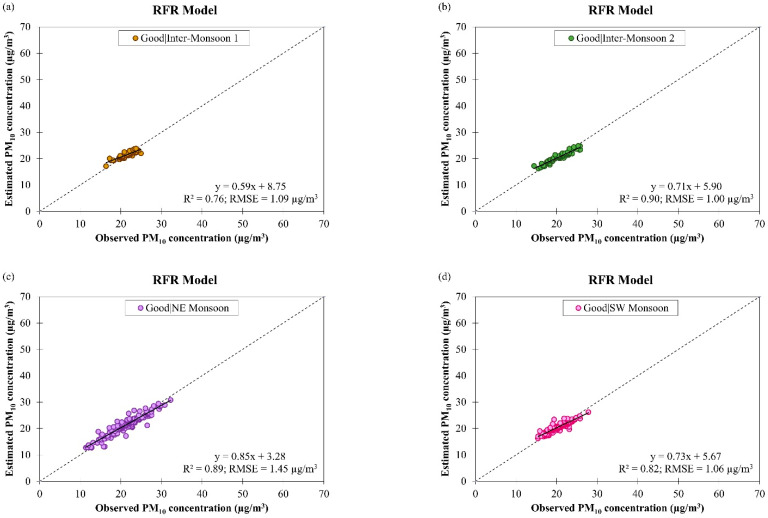

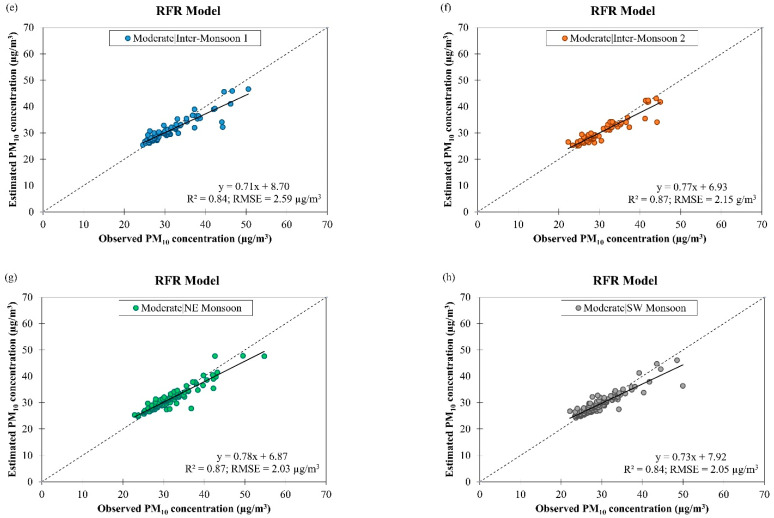
Scatter plots of estimated daily PM_10_ concentration against observed daily PM_10_ concentration by RFR model for (**a**–**d**) good and (**e**–**h**) moderate air quality conditions during different monsoon seasons in Singapore from March 2016 to February 2018.

Scatter plots of the observed and estimated daily PM_10_ concentrations by the RFR model for good and moderate air quality conditions during different monsoon seasons in Singapore from March 2016 to February 2018 are shown in Figure 12. As the air quality changed from good to moderate, the fitting of data on the RFR model was improved (as indicated by an increment in the $R^{2}$ value from 0.84 to 0.86, on average) but the accuracy of the model was reduced (as indicated by an increment in the RMSE value from 1.15 µg/m^3^ to 2.21 µg/m^3^, on average). The accuracy of the RFR model can possibly be increased by increasing the number of trees in future studies. The RFR model for daily PM_10_ concentration estimation in different monsoon seasons in Singapore has an $R^{2}$ value ranging between 0.76 and 0.90 during good air quality and between 0.84 and 0.87 during moderate air quality with RMSE value ranging between 1.00 µg/m^3^ and 1.45 µg/m^3^ during good air quality and between 2.03 µg/m^3^ and 2.59 µg/m^3^ during moderate air quality. The performances of the ANCOVA and RFR models for daily PM_10_ concentration estimation during good and moderate air quality in different monsoon seasons in Singapore were compared in Table 3 and the results show that the RFR model was more accurate with better data fitting ($R^{2}$ = 0.85 and RMSE = 1.68 µg/m^3^, on average) than the ANCOVA model ($R^{2}$ = 0.50 and RMSE = 2.83 µg/m^3^, on average).

### 3.4. Application of the Derived Models for Estimating PM_2.5_ and PM_10_ Concentrations in Brunei Darussalam

To test the applicability of the derived models as cross-country models for estimating PM_2.5_ and PM_10_ concentrations in Southeast Asia, they were tested with air quality and meteorological data from Brunei Darussalam from January 2009 to December 2019 as an example. The results for PM_2.5_ and PM_10_ concentrations estimations in Brunei Darussalam were provided and discussed separately in the following sections.

#### 3.4.1. Estimation of PM_2.5_ Concentration in Brunei Darussalam

**Figure 13:**
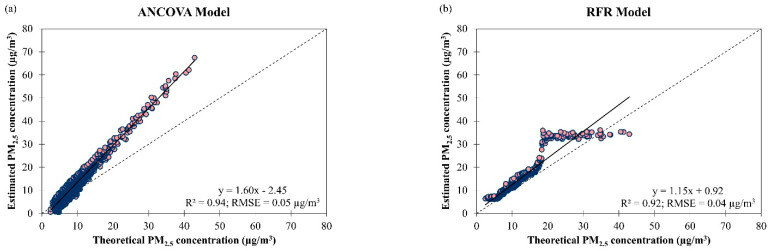
Scatter plots of estimated daily PM_2.5_ concentration against theoretical daily PM_2.5_ concentration by (**a**) ANCOVA and (**b**) RFR models for overall air quality in Brunei Darussalam from January 2009 to December 2019.

Figure 13 shows that there was no major difference in terms of data fitting and accuracy between the ANCOVA ($R^{2}$ = 0.94 and RMSE = 0.05 µg/m^3^) and RFR models ($R^{2}$ = 0.92 and RMSE = 0.04 µg/m^3^) when they were used to estimate daily PM_2.5_ concentration in Brunei Darussalam. Both models yielded better data fitting and accuracy for estimating daily PM_2.5_ concentration in Brunei Darussalam ($R^{2}$ = 0.93 and RMSE = 0.05 µg/m^3^, averaged between both models) compared to Singapore ($R^{2}$ = 0.82 and RMSE = 0.09 µg/m^3^, averaged between both models). Based on the RMSE value, both ANCOVA and RFR models for estimating daily PM_2.5_ concentration from March 2016 to February 2018 (2 years) in Singapore (RMSE = 0.10 µg/m^3^ for the ANCOVA model and 0.07 µg/m^3^ for the RFR model) and from 2009 to 2019 (11 years) in Brunei Darussalam (RMSE = 0.05 µg/m^3^ for the ANCOVA model and 0.04 µg/m^3^ for the RFR model) presented in this study outperformed the long short-term memory (LSTM) model (RMSE = 8.91 µg/m^3^) for estimating 48-h PM_2.5_ concentration from 2013 to 2016 (4 years) in Iran in 2019 [46] and the gradient boosting model (RMSE = 7.06 µg/m^3^) for estimating 24-h PM_2.5_ concentration for the year 2017 (1 year) in Taiwan in 2020 [47]. These could be attributed to more homogeneous topography (mainly basins) in Singapore and Brunei Darussalam compared to complicated topography in Iran and Taiwan (with mountainous layouts), leading to the simpler dispersal of air pollutants.

A stagnant pattern of PM_2.5_ concentration at theoretical values beyond 18 µg/m^3^ was seen in Figure 13b and this shows that the derived RFR model was not able to accurately estimate daily PM_2.5_ concentration in Brunei Darussalam beyond this concentration. This could be due to insufficient explanatory variables [21,48] to describe the increase in PM_2.5_ concentration in Brunei Darussalam that could be attributed to the occurrence of smoke haze. A possible explanatory variable that could be added to the proposed models is wildfires information because the occurrence of intense wildfires will lead to a heatwave and high PM_2.5_ concentrations that may be transported thousands of kilometers away from their source areas [48,49], affecting the air quality in the nearby regions. For example, long-ranged transported PM pollution episodes caused by wildfires in eastern Europe (Russia, Belarus, Ukraine and the Baltic countries) are common in Finland [50]. The study found that the ANCOVA model underestimated by 17% (17% of the observations) and overestimated by 32% (83% of the observations) on the daily PM_2.5_ concentration in Brunei Darussalam. Meanwhile, the RFR model tends to overestimate the daily PM_2.5_ concentration in Brunei Darussalam by 30% (99.7% of the observations) and underestimated by 8% (0.3% of the observations). The best estimation for PM_2.5_ concentration in Brunei Darussalam was at 4.08 µg/m^3^ for the ANCOVA model but that could not be determined for the RFR model (Figure 13).

**Figure 14:**
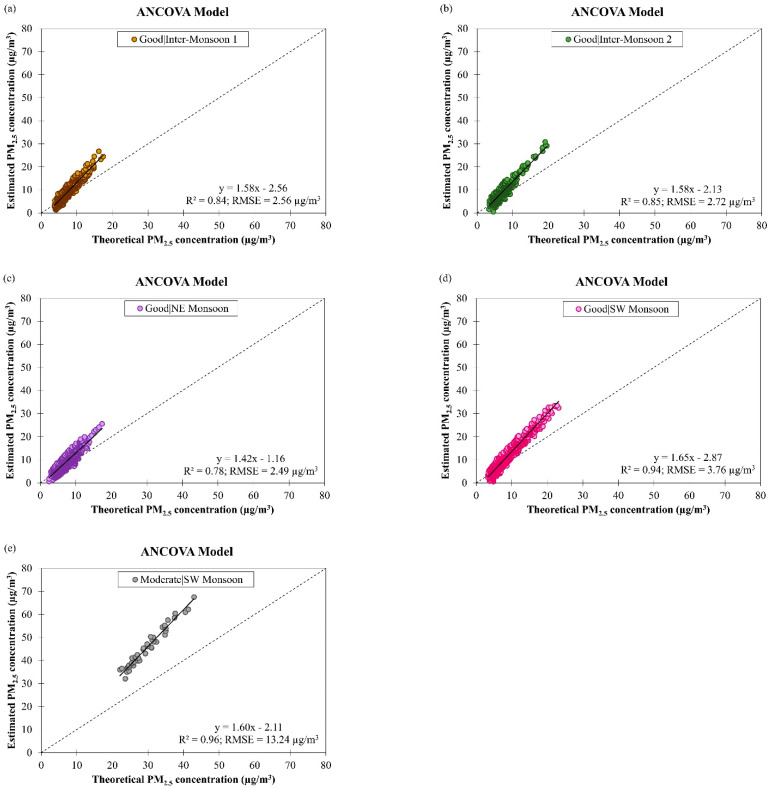
Scatter plots of estimated daily PM_2.5_ concentration against theoretical daily PM_2.5_ concentration by ANCOVA model for (**a**–**d**) good air quality condition during different monsoon seasons and (**e**) moderate air quality condition during SW monsoon in Brunei Darussalam from January 2009 to December 2019.

There are no scatter plots presented in this section to show the estimation results of the derived ANCOVA and RFR models for moderate air quality in NE and both inter-monsoon seasons in Brunei Darussalam because of the limited availability of theoretical PM_2.5_ and observed PM_10_ concentrations data during these air quality conditions and monsoon seasons. The estimation results of the derived ANCOVA model for daily PM_2.5_ concentration for good and moderate air quality conditions during other monsoon seasons in Brunei Darussalam from January 2009 to December 2019 are shown in Figure 14. When the good air quality became moderate, the accuracy of the model was worsened (as indicated by a significant increment in the RMSE value from 2.88 µg/m^3^ to 13.24 µg/m^3^, on average) despite improvement in data fitting (as indicated by an increment in the $R^{2}$ value from 0.85 to 0.96, on average). This could possibly be due to the inadequate interaction level among the model’s input parameters being accounted for by the derived ANCOVA model. Table 5 shows that the $R^{2}$ value of the ANCOVA model for estimating daily PM_2.5_ concentration in different monsoon seasons in Brunei Darussalam was ranged between 0.78 and 0.94 during good air quality and 0.96 during moderate air quality, and the RMSE value was ranged between 2.49 µg/m^3^ and 3.76 µg/m^3^ during good air quality and 13.24 µg/m^3^ during moderate air quality. The highest RMSE value (13.24 µg/m^3^) was obtained during moderate air quality in SW monsoon season, and this could be due to the large variation in daily PM_2.5_ concentration (see Figure 4a) that was likely to be contributed by the smoke haze event occurring in this season. The ANCOVA model for daily PM_2.5_ concentration in Brunei Darussalam showed the best performance during NE monsoon season at good air quality condition by having the highest estimation accuracy (RMSE = 2.49 µg/m^3^) despite having a trade-off with the fitting of the data (as indicated by the lowest $R^{2}$ value of 0.78).

**Figure 15:**
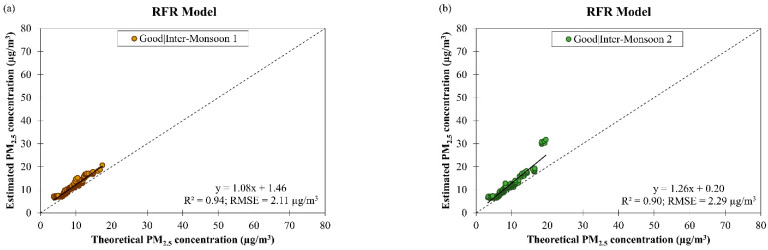

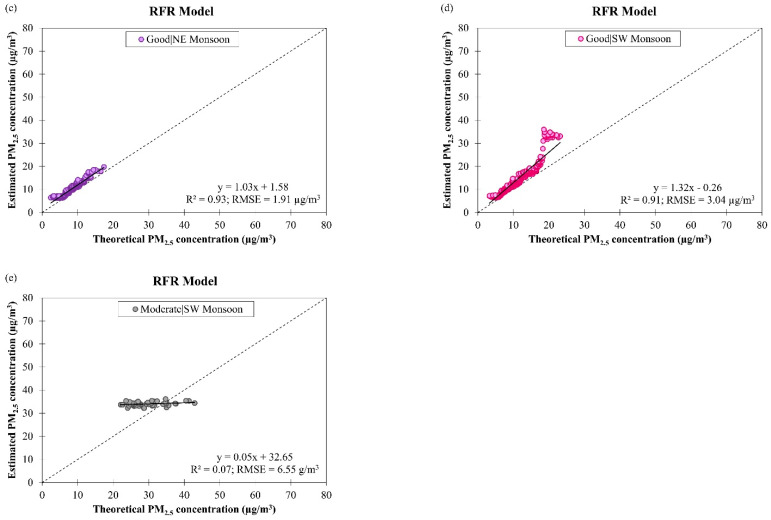
Scatter plots of estimated daily PM_2.5_ concentration against theoretical daily PM_2.5_ concentration by RFR model for (**a**–**d**) good air quality condition during different monsoon seasons and (**e**) moderate air quality during SW monsoon in Brunei Darussalam from January 2009 to December 2019.

Figure 15 shows the estimation results of the derived RFR model for daily PM_2.5_ concentration for good and moderate air quality conditions during different monsoon seasons in Brunei Darussalam from January 2009 to December 2019. The fitting of data on the RFR model was worsened (as indicated by a major drop in the $R^{2}$ value from 0.92 to 0.07, on average) and the accuracy of the model was decreased (as indicated by an increment in the RMSE value from 2.34 µg/m^3^ to 6.55 µg/m^3^, on average) when the air quality changed from good to moderate. The ranges of $R^{2}$ value of the RFR model for estimating daily PM_2.5_ concentration in different monsoon seasons in Brunei Darussalam were between 0.90 and 0.94 during good air quality and 0.07 during moderate air quality, and the ranges of RMSE value of the RFR model were between 1.99 µg/m^3^ and 3.04 µg/m^3^ during good air quality and 6.55 µg/m^3^ during moderate air quality. The comparison of the performances of both models for daily PM_2.5_ concentration estimation during good and moderate air quality conditions in different monsoon seasons in Brunei Darussalam are shown in Table 5 and the results show that the derived ANCOVA model generally gave better fitting on the data although it has a slightly lower accuracy ($R^{2}$ = 0.87 and RMSE = 4.95 µg/m^3^, on average) than the RFR model ($R^{2}$ = 0.75 and RMSE = 3.18 µg/m^3^, on average). However, having said that, both derived models show limitations in handling high PM_2.5_ concentrations when tested on the datasets from both countries; therefore, further studies to improve these models are necessary before they can be used as cross-country models.

#### 3.4.2. Estimation of PM_10_ Concentration in Brunei Darussalam

**Figure 16:**
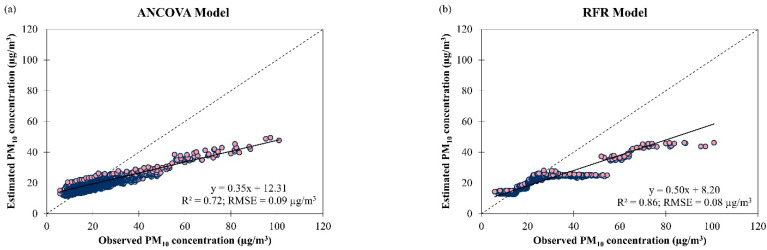
Scatter plots of estimated daily PM_10_ concentration against observed daily PM_10_ concentration by (**a**) ANCOVA and (**b**) RFR models for overall air quality in Brunei Darussalam from January 2009 to December 2019.

The testing results of the derived ANCOVA and RFR models to estimate daily PM_10_ concentrations in Brunei Darussalam from January 2009 to December 2019 are presented in Figure 16. It was seen that the RFR model has comparable data fitting and accuracy ($R^{2}$ = 0.86 and RMSE = 0.08 µg/m^3^) compared to the ANCOVA model ($R^{2}$ = 0.723 and RMSE = 0.09 µg/m^3^) although the RFR model could not handle observed PM_10_ concentration over 18 µg/m^3^ (as indicated by the stagnant estimated PM_10_ concentrations in Figure 16b). Additional input parameters such as vehicular traffic and forest fires information need to be considered to improve the estimation performance of the derived models. Due to the low RMSE value of both derived models, the ANCOVA model underestimated and overestimated the daily PM_10_ concentration in Brunei Darussalam by 1% (50% of the observations) and the RFR model underestimated by 9% (54% of the observations) and overestimated by 14% (46% of the observations). The best estimation of PM_10_ concentration in Brunei Darussalam was at 18.94 µg/m^3^ for the ANCOVA model and 16.40 µg/m^3^ for the RFR model (Figure 16).

Both ANCOVA and RFR models for estimating daily PM_10_ concentration from March 2016 to February 2018 (2 years) in Singapore (RMSE = 0.11 µg/m^3^ for the ANCOVA model and 0.07 µg/m^3^ for the RFR model) and from January 2009 to December 2019 (11 years) in Brunei Darussalam (RMSE = 0.09 µg/m^3^ for the ANCOVA model and 0.08 µg/m^3^ for the RFR model) showed better estimation performance than the MLR model (RMSE = 126.73 µg/m^3^) for estimating hourly PM_10_ concentration during transboundary haze events from 2005 to 2014 (excluding the years from 2007 to 2009) (7 years) in Sarawak and Peninsular Malaysia in 2020 [51]. This could be due to the spatial and temporal variations of PM emissions from other major air pollution sources such as motor vehicles and industrial activities as a result of different developments among countries in Southeast Asia [52]. For example, in Singapore, motor vehicles account for about 50% of the total PM_2.5_ emissions [53] and about 60% of the estimated total ground transportation PM emissions [54]. In Malaysia, motor vehicles contributed to about 17% of the total PM emissions in 2010 [55] and the highest mean monthly PM_10_ concentration (68.79 µg/m^3^) between 1997 and 2015 was recorded in Port Klang, Malaysia [56] due to high traffic volume and proportion of diesel vehicles [57].

**Figure 17:**
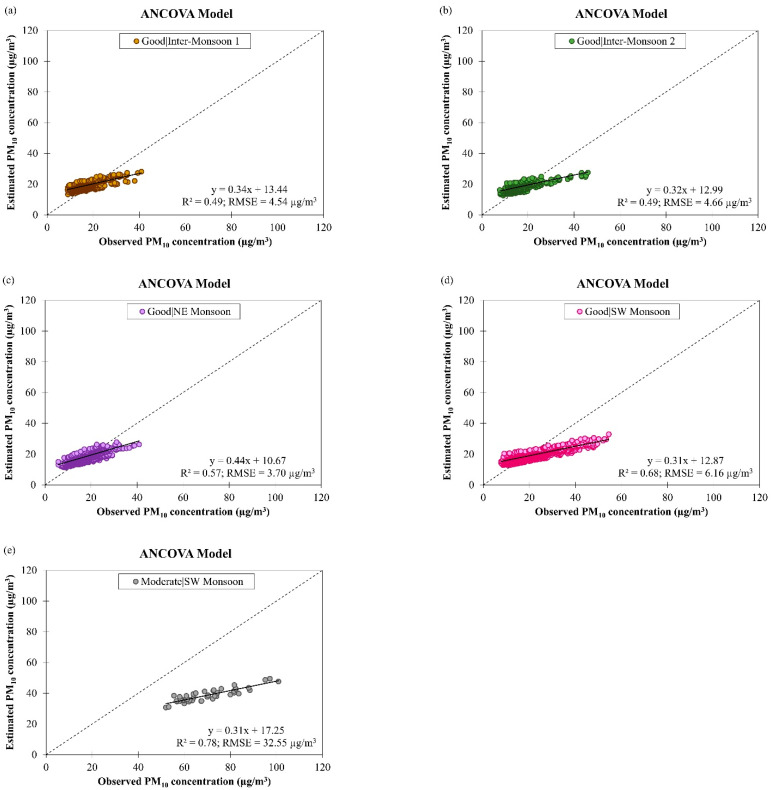
Scatter plots of estimated daily PM_10_ concentration against observed daily PM_10_ concentration by ANCOVA model for (**a**–**d**) good air quality condition during different monsoon seasons and (**e**) moderate air quality condition during SW monsoon in Brunei Darussalam from January 2009 to December 2019.

Figure 17 shows the observed and estimated daily PM_10_ concentrations by the ANCOVA model for good and moderate air quality conditions during different monsoon seasons in Brunei Darussalam from January 2009 to December 2019. It can be seen that the accuracy of the derived ANCOVA model was reduced (as indicated by an increment in the RMSE value from 19.06 µg/m^3^ to 32.55 µg/m^3^, on average) despite better data fitting on the model (as indicated by an increment in the $R^{2}$ value from 0.56 to 0.78, on average) when the air quality changed from good to moderate. This may imply that the confounding effects between the input parameters of the model were not well described by the model. To overcome this, the interaction level of the ANCOVA model should be increased in future studies. Table 5 shows that the derived ANCOVA model for estimating daily PM_10_ concentration in different monsoon seasons in Brunei Darussalam has $R^{2}$ value ranging between 0.49 and 0.68 during good air quality and 0.78 during moderate air quality with RMSE value ranging between 3.70 µg/m^3^ and 6.16 µg/m^3^ during good air quality and 32.55 µg/m^3^ during moderate air quality. The highest RMSE value (32.55 µg/m^3^) was obtained during moderate air quality in SW monsoon season due to the large variation in daily PM_10_ concentration (see Figure 4b) that could have resulted from the occurrence of transported smoke haze in the region during this season.

**Figure 18:**
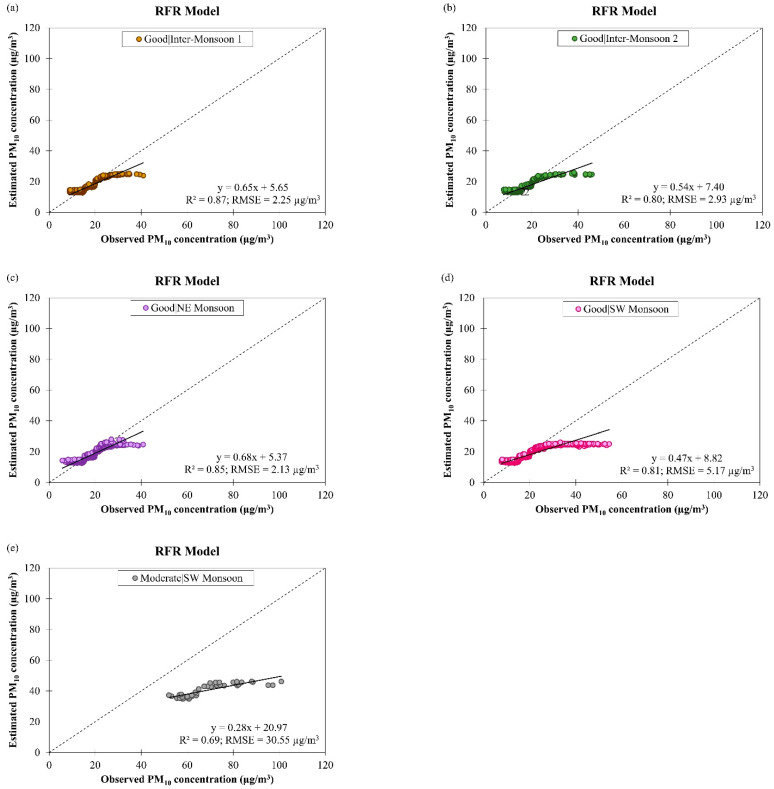
Scatter plots of estimated daily PM_10_ concentration against observed daily PM_10_ concentration by RFR model for (**a**–**d**) good air quality condition during different monsoon seasons and (**e**) moderate air quality condition during SW monsoon in Brunei Darussalam from January 2009 to December 2019.

The performance of the derived RFR model for estimating daily PM_10_ concentration for good and moderate air quality conditions during different monsoon seasons in Brunei Darussalam from January 2009 to December 2019 are shown in Figure 18. As the air quality became moderate, the data fitting and accuracy of the RFR model were reduced (as indicated by the drop in the $R^{2}$ value from 0.83 to 0.69, on average, and the increase in the RMSE value from 3.12 µg/m^3^ to 30.55 µg/m^3^, on average). This showed that the derived RFR model could not describe the increase in PM_10_ concentration well. When the derived RFR model was used to estimate daily PM_10_ concentration estimation in different monsoon seasons in Brunei Darussalam, it gave $R^{2}$ value between 0.80 and 0.87 during good air quality and 0.69 during moderate air quality with RMSE value between 2.13 µg/m^3^ and 5.17 µg/m^3^ during good air quality and 30.55 µg/m^3^ during moderate air quality. Although the derived ANCOVA model generally fits the data less well and it yielded lower accuracy ($R^{2}$ = 0.60 and RMSE = 10.32 µg/m^3^, on average) compared to the RFR model ($R^{2}$ = 0.80 and RMSE = 8.61 µg/m^3^, on average) (Table 5), the derived ANCOVA model can handle increased PM_10_ concentration much better than the RFR model, particularly during moderate air quality in SW monsoon season.

## 4. Conclusions

This study explores the potential to estimate daily PM_2.5_ and PM_10_ concentrations in Brunei Darussalam using ML-based statistical models (such as ANCOVA and RFR) derived from air quality and meteorological data in Singapore with statistical assessments. The most influential explanatory variables for estimating PM_2.5_ and PM_10_ concentrations in Singapore by the ANCOVA model were the interaction variables (PM_10_ concentration × air quality condition) and (wind direction × air quality condition), respectively. Meanwhile, the most important variables for estimating daily PM_2.5_ and PM_10_ concentrations in Singapore by the RFR model were PM_10_ concentration and air quality condition, respectively. Both ANCOVA ($R^{2}$ = 0.75 and RMSE = 0.10 µg/m^3^ for PM_2.5_, and $R^{2}$ = 0.81 and RMSE = 0.11 µg/m^3^ for PM_10_) and RFR models ($R^{2}$ = 0.89 and RMSE = 0.07 µg/m^3^ for PM_2.5_, and $R^{2}$ = 0.93 and RMSE = 0.07 µg/m^3^ for PM_10_) performed well when used to estimate daily PM_2.5_ and PM_10_ concentrations in Singapore. When these derived models were tested with air quality and meteorological data from Brunei Darussalam to estimate its daily PM_2.5_ and PM_10_ concentrations, the ANCOVA model seems to perform better ($R^{2}$ = 0.94 and RMSE = 0.05 µg/m^3^ for PM_2.5_, and $R^{2}$ = 0.72 and RMSE = 0.09 µg/m^3^ for PM_10_) than the RFR model ($R^{2}$ = 0.92 and RMSE = 0.04 µg/m^3^ for PM_2.5_, and $R^{2}$ = 0.86 and RMSE = 0.08 µg/m^3^ for PM_10_) because it can describes PM concentrations over 18 µg/m^3^ better than the RFR model.

The limitations of the models in this work are due to insufficient data for unique incidents (for example, serious smoke haze leading to moderate or unhealthy air quality condition), low interaction level among the ANCOVA model’s input parameters used in the model development, low number of trees when developing the RFR model and insufficient explanatory variables relating to atmospheric PM pollution (for examples, vehicular traffic and forest fires information). These estimation models for PM concentrations can be improved further in future studies by including more data recorded during moderate and/or unhealthy air quality conditions, increasing the interaction level of the ANCOVA model, increasing the number of trees for the RFR model, performing cross-validation on the datasets and including major domestic anthropogenic emissions such as vehicular traffic and/or forest fire information as the explanatory variables. Overall, the study had demonstrated the potential of applying the models as cross-country models in the Southeast Asia region although more actual/measured PM_2.5_ concentration data from Brunei Darussalam in the future are needed to test the accuracy of the models and more experimental on fine-tuning the models’ parameters to improve the model performance as well as their capability in handling higher PM concentrations in the region.

## Figures and Tables

**Table 1:** Descriptive statistics of measured quantitative variables from all monitoring areas.

Country	Period	Variable	Minimum	Maximum	Mean	Standard Deviation
Singapore	March 2016 to February 2018 (2 years)	Observed PM_2.5_ (µg/m^3^)	4.38	63.84	14.46	5.39
		Observed PM_10_ (µg/m^3^)	11.38	54.80	26.00	6.71
		Air temperature, T (°C)	22.51	30.27	27.53	1.16
		Wind speed, WS (m/s)	0.50	5.98	2.21	0.85
		Wind direction, WD (°)	17.63	332.85	140.39	67.13
		Rainfall, R (mm)	0	5.32	0.32	0.66
Brunei Darussalam	January 2009 to December 2019 (11 years)	Theoretical PM_2.5_ (µg/m^3^)	2.45	42.93	7.67	3.73
		Observed PM_10_ (µg/m^3^)	5.75	100.90	18.02	8.76
		Air temperature, T (°C)	23.20	31.00	27.79	1.03
		Wind speed, WS (m/s)	1.05	7.05	2.35	0.62
		Wind direction, WD (°)	1.96	33.13	20.43	3.59
		Rainfall, R (mm)	0	11.46	0.37	0.79

**Table 2:** Results of sum of squares of the errors (SSE) analysis on the ANCOVA model for estimating daily PM_2.5_ concentration in Singapore from March 2016 to February 2018 for selected explanatory variables.

Explanatory Variable	Degree of Freedom (DF)	Sum of Squares of the Errors (SSE) (µg/m^3^)	Mean Squared Error (MSE) (µg/m^3^)	F Value	Probability > F Value
Day × Air quality condition	2	52.54	26.27	3.53	0.03
Year × Wind speed	1	6.32	6.32	0.85	0.36
Year × Air quality condition	2	193.05	96.53	12.95	3.16 × $10^{-6}$
Air temperature × Wind speed	1	3.79	3.79	0.51	0.48
Air temperature × Wind direction	1	0	0	<0.0001	1
Air temperature × PM_10_	1	16.59	16.59	2.23	0.14
Wind direction × PM_10_	1	0	0	<0.0001	1
PM_10_ × Air quality condition	1	171.98	171.98	23.08	2.00 × $10^{-6}$

**Table 3:** Comparison of determination coefficient ($R^{2}$ ) and root mean square of the errors (RMSE) of the ANCOVA and RFR models for estimating daily PM_2.5_ and PM_10_ concentrations during good and moderate air quality conditions in different monsoon seasons in Singapore from March 2016 to February 2018.

Air Quality Condition|Monsoon Season	Statistical Indicator	PM_2.5_		PM_10_	
		ANCOVA Model	RFR Model	ANCOVA Model	RFR Model
Overall	$R^{2}$	0.75	0.89	0.81	0.93
	RMSE (µg/m^3^)	0.10	0.07	0.11	0.07
Good|Inter-Monsoon 1	$R^{2}$	0.20	0.74	0.18	0.76
	RMSE (µg/m^3^)	1.95	1.13	2.19	1.09
Good|Inter-Monsoon 2	$R^{2}$	0.39	0.88	0.38	0.90
	RMSE (µg/m^3^)	1.95	0.96	2.20	1.00
Good|NE Monsoon	$R^{2}$	0.61	0.85	0.63	0.89
	RMSE (µg/m^3^)	1.58	1.02	2.65	1.45
Good|SW Monsoon	$R^{2}$	0.35	0.76	0.38	0.82
	RMSE (µg/m^3^)	1.65	1.01	1.93	1.06
Moderate|Inter-Monsoon 1	$R^{2}$	0.61	0.72	0.56	0.84
	RMSE (µg/m^3^)	3.19	2.85	3.98	2.59
Moderate|Inter-Monsoon 2	$R^{2}$	0.78	0.85	0.76	0.87
	RMSE (µg/m^3^)	2.43	2.15	2.86	2.15
Moderate|NE Monsoon	$R^{2}$	0.57	0.77	0.61	0.87
	RMSE (µg/m^3^)	2.83	2.05	3.46	2.03
Moderate|SW Monsoon	$R^{2}$	0.50	0.87	0.53	0.84
	RMSE (µg/m^3^)	4.08	2.09	3.38	2.05

**Table 4:** Results of sum of squares of the errors (SSE) analysis on the ANCOVA model for estimating daily PM_2.5_ concentration in Singapore from March 2016 to February 2018 for selected explanatory variables.

Explanatory Variable	Degree of Freedom (DF)	Sum of Squares of the Errors (SSE) (µg/m^3^)	Mean Squared Error (MSE) (µg/m^3^)	F Value	Probability > F
Year × Air temperature	1	0	0	<0.0001	1
Year × Wind speed	1	82.77	82.77	10.38	1.35 × $10^{-3}$
Year × PM_2.5_	1	38.96	38.96	4.89	0.03
Air temperature × Wind speed	1	2.08	2.08	0.26	0.61
Air temperature × PM_2.5_	1	145.60	145.60	18.26	2.26 × $10^{-5}$
Wind speed × PM_2.5_	1	62.66	62.66	7.86	5.23 × $10^{-3}$
Wind speed × Air quality condition	2	182.57	91.29	11.45	1.34 × $10^{-5}$
Wind direction × PM_2.5_	1	156.94	156.94	19.69	1.10 × $10^{-5}$
Wind direction × Air quality condition	2	204.49	102.25	12.83	3.58 × $10^{-6}$
PM_2.5_ × Monsoon season	3	97.09	32.36	4.06	7.19 × $10^{-3}$

**Table 5:** Comparison of determination coefficient ($R^{2}$ ) and root mean square of the errors (RMSE) of the ANCOVA and RFR models for estimating daily PM_2.5_ and PM_10_ concentrations during good and moderate air quality conditions in different monsoon seasons in Brunei Darussalam from January 2009 to December 2019.

Air Quality Condition|Monsoon Season	Statistical Indicator	PM_2.5_		PM_10_	
		ANCOVA Model	RFR Model	ANCOVA Model	RFR Model
Overall	$R^{2}$	0.94	0.92	0.72	0.86
	RMSE (µg/m^3^)	0.05	0.04	0.09	0.08
Good|Inter-Monsoon 1	$R^{2}$	0.84	0.94	0.49	0.87
	RMSE (µg/m^3^)	2.56	2.11	4.54	2.25
Good|Inter-Monsoon 2	$R^{2}$	0.85	0.90	0.49	0.80
	RMSE (µg/m^3^)	2.72	2.29	4.66	2.93
Good|NE Monsoon	$R^{2}$	0.78	0.93	0.57	0.85
	RMSE (µg/m^3^)	2.49	1.91	3.70	2.13
Good|SW Monsoon	$R^{2}$	0.94	0.91	0.68	0.81
	RMSE (µg/m^3^)	3.76	3.04	6.16	5.17
Moderate|SW Monsoon	$R^{2}$	0.96	0.07	0.78	0.69
	RMSE (µg/m^3^)	13.24	6.55	32.55	30.55

## Data Availability

The air quality and meteorological data in Singapore are available at https://www.haze.gov.sg/resources/pollutant-concentrations (accessed on 15 January 2020) and https://www.nusurbanclimate.com/weather-portal (accessed on 17 February 2020). The air quality and meteorological data in Brunei Darussalam are not publicly available because restrictions apply to the availability of these data that were used under license for this study.
